# The Landscape of Genetic Alterations Stratified Prognosis in Oriental Pancreatic Cancer Patients

**DOI:** 10.3389/fonc.2021.717989

**Published:** 2021-07-22

**Authors:** Shiwei Guo, Xiaohan Shi, Suizhi Gao, Qunxing Hou, Lisha Jiang, Bo Li, Jing Shen, Huan Wang, Shuo Shen, GuoXiao Zhang, Yaqi Pan, Wuchao Liu, Xiongfei Xu, Kailian Zheng, Zhuo Shao, Wei Jing, Ling Lin, Gang Li, Gang Jin

**Affiliations:** ^1^Department of Hepatobiliary Pancreatic Surgery, Changhai Hospital, Naval Military Medical University (Second Military Medical University), Shanghai, China; ^2^Department of General Surgery, Naval Medical Center of People's Liberation Army (PLA), Shanghai, China; ^3^Zhangjiang Center for Translational Medicine, Shanghai Biotecan Medical Diagnostics Co., Ltd, Shanghai, China

**Keywords:** pancreatic ductal adenocarcinoma, clinicopathological variable, network-based stratification, prognosis, genetic alteration

## Abstract

**Background:**

Pancreatic cancer is a life-threatening malignant disease with significant diversity among geographic regions and races leading to distinct carcinogenesis and prognosis. Previous studies mainly focused on Western patients, while the genomic landscape of Oriental patients, especially Chinese, remained less investigated.

**Methods:**

A total of 408 pancreatic cancer patients were enrolled. A panel containing 436 cancer-related genes was used to detect genetic alterations in tumor samples.

**Results:**

We profiled the genomic alteration landscape of pancreatic duct adenocarcinoma (PDAC), intraductal papillary mucinous neoplasm (IPMN), periampullary carcinoma (PVC), and solid-pseudopapillary tumor (SPT). Comparison with a public database revealed specific gene mutations in Oriental PDAC patients including higher mutation rates of DNA damage repair-related genes. Analysis of mutational signatures showed potential heterogenous carcinogenic factors caused by diabetes mellitus. KRAS mutation, especially KRAS G12D mutation, was associated with poor survival, while patients not harboring the 17 significant copy number variations (CNVs) had a better prognosis. We further identified multiple correlations between clinicopathologic variables and genetic mutations, as well as CNVs. Finally, by network-based stratification, three classes of PDAC patients were robustly clustered. Among these, class 1 (characterized by the Fanconi anemia pathway) achieved the best outcome, while class 2 (involved in the platinum drug resistance pathway) suffered from the worst prognosis.

**Conclusions:**

In this study, we reported for the first time the genetic alteration landscape of Oriental PDAC patients identifying many Oriental-specific alterations. The relationship between genetic alterations and clinicopathological factors as well as prognosis demonstrated important genomic impact on tumor biology. This study will help to optimize clinical treatment of Oriental PDAC patients and improve their survival.

## Introduction

Pancreatic cancer is a devastating malignant disease with a median survival of 6–12 months and a 5-year survival rate of less than 9% (1, 2). Although the prognosis is very poor worldwide, heterogeneity exists between different regions and races due to lifestyle behaviors and environmental factors (3). Large differences in tobacco intake, dietary habits, and diabetes mellitus between the East and the West may lead to different genomic alterations associated with pancreatic carcinogenesis (4–6), which might cause variation in chemosensitivity and the effectiveness of drugs in Oriental patients. Current studies are mainly based on Western patients, and there is a lack of comparative studies on Oriental pancreatic cancer patients, especially among the largest patient population in China.

Gene mutations are the primary cause of cancer. Specific germline or genetic driver mutations can initialize the process of carcinogenesis (7). Diverse combinations of genetic drivers and passenger mutations (8) result from different carcinogenic factors. For example, high glucose can lead to heterogeneity in pancreatic cancer (9). Furthermore, heterogeneity brings significant differences in biological behaviors of pancreatic cancer, including malignant proliferation (10), metastasis potential (11), and chemotherapy sensitivity (12), and these differences can affect prognosis (13, 14). Therefore, defining the mutation landscape of pancreatic cancer in Oriental patients by comparing the characteristics between Eastern and Western patients may aid in identifying possible causes of differences in pathogenesis and prognosis.

Since the dimensions of genetic variation far exceed clinical information, effectively elucidating the relationship between genetic alterations, clinical pathology, and prognosis remains a great challenge. Conventional panel (15, 16) or exon sequencing (17, 18) usually identified hundreds of genetic alterations including single nucleotide variation (SNV), insertion and deletion (InDel), and copy number variation (CNV) information. Such multidimensional data often resulted in over-fitting in correlation or prognosis analysis. Therefore, efficient data dimension reduction methods and large-scale patient data are both necessary. In this study, we utilized the high-quality prospective database of the Changhai Hospital and a variety of dimension reduction methods to explore the relationship between genetic alterations and clinicopathological factors, as well as patient prognosis for revealing the characteristics of pancreatic cancer in the Oriental population.

## Materials and Methods

### Patients

Patients who underwent radical resection for peripancreatic lesions and received preoperative or intraoperative biopsy (fine-needle aspiration biopsy, FNA) in the Department of Hepatobiliary Pancreatic Surgery of the Changhai Hospital (Shanghai, China) between 2016 and 2018 were enrolled in this study. Exclusion criteria included insufficient tissue for sequencing and use of neoadjuvant therapy. Patients with incomplete follow-up data and 90-day postoperative mortality were further excluded from the survival analysis. All of the patients provided written informed consent for use of their clinical data. The study was conducted in accordance with national guidelines and approved by the Ethics Committee of the Changhai Hospital.

### Clinical Samples

After the isolation of samples, tumor tissues were quickly frozen in liquid nitrogen and transferred to the laboratory. The surgical tissue was continuously sectioned on a frozen edge-cutting machine and then stained with H&E to confirm tumor cellularity. The remaining surgical tissues or biopsy samples were used for DNA extraction. The final pathological diagnosis was confirmed by two independent pathologists. Finally, four subtypes of neoplasm-pancreatic ductal adenocarcinoma, periampullary carcinoma, intraductal papillary mucinous neoplasm, and solid-pseudopapillary tumors were included in the following analysis.

### DNA Extraction and Quality Control

DNA was extracted from fresh tissue using the QIAamp DNA Mini Kit (QIAGEN, CA, USA). DNA quantity and purity were assessed by Qubit^®^ 3.0 Fluorometer (Invitrogen, Carlsbad, CA, USA) and NanoDrop ND-1000 (Thermo Scientific, Wilmington, DE, USA). Fragmentation status was evaluated *via* the Agilent 2200 TapeStation system using the Genomic DNA ScreenTape assay (Agilent Technologies, Santa Clara, CA, USA) able to produce a DNA Integrity Number (DIN).

### Library Preparation and Sequencing

A total of 500 ng of DNA per sample was used for the DNA library preparation. The library was generated using the Agilent SureSelect XT HS (Agilent Technologies) according to the manufacturer’s instructions. First, DNA was fragmented on an E220 focused ultrasonicator Covaris (Covaris, Woburn, MA, USA) to a size of 150–220 bp. Then, the DNA fragments were end-polished, A-tailed, and ligated with adaptors. The exons of 436 cancer-related genes were captured (**Supplementary Table 1**) and amplified *via* PCR. After QC and quantification using an Agilent 2100 Bioanalyzer (Agilent Technologies) and Qubit^®^ 3.0 Fluorometer (Invitrogen), sequencing was performed using an Illumina Next CN500 platform (Berry Genomics) by paired-end 75-bp or 150-bp to a mean unique depth of coverage of 800X.

### Bioinformatics Analysis

Sequencing data were mapped to the reference human genome (UCSC hg19) *via* Burrows-Wheeler Aligner (BWA) software to obtain the original mapping results stored in a BAM format (19, 20). Then, SAMtools was used to sort the BAM files and perform duplicate marking, local realignment, and base quality recalibration to generate the final BAM file for computing the sequence coverage and depth (21). GATK4 Mutect2 is the favored strategy for tumor-only generation of a complete list of somatic mutations, including SNV and InDel (22, 23). Briefly, we created a panel of normal resources using the tumor-only mode of Mutect2, generated the genetic mutations, and filtered the callset with FilterMutectCalls. ANNOVAR was performed to annotate the variant call format file obtained in the previous step (24). The mutations with a variant allele frequency > 5% were defined as high confidence mutations. Tumor mutation load was defined as the number of all genetic SNVs and InDels per mega base, excluding synonymous mutations.

The mutational signature was analyzed using R software and the Maftools package (25). The pattern of the mutation signature was distributed into six substitution classes, and the bases immediately 5′ and 3′ of the mutated base produced 96 possible mutation subtypes (26, 27). The mutation signature found in the tumors was analyzed by non-negative matrix factorization (NMF) and compared to 30 types of mutational signatures (28). CNV was identified utilizing a CNVkit and GISTIC2.0 (29, 30). False discovery rate q-values for the aberrant regions (0.25 as the threshold) and G-score, considering the amplitude of the aberration as well as the frequency of its occurrence across samples, were calculated with GISTIC2.0. Network-based stratification was performed to produce a robust subdivision of patients into classes (31).

### Clinical Data and Follow-Up

Demographic and clinicopathological data were extracted from the prospective database of the Changhai Hospital. Preoperative clinical variables included sex, age, body mass index (BMI), smoking history, drinking history, diabetes mellitus, hypertension, carcinoembryonic antigen (CEA), and carbohydrate antigen 19-9 (CA19-9). Postoperative variables included differentiation degree, perineural invasion, and microvascular invasion as well as pathological T, N, and M stages (the eighth edition of American Joint Committee on Cancer). Patients were followed up every 3 months by telephone after surgery or biopsy. Overall survival (OS) was defined as the interval from the date of surgery or biopsy until the date of patient death or the last follow-up visit after 3 months post-surgery.

### Statistical Analysis

Discrete variables were presented as number and/or percentage, whereas the continuous variables were presented as median and/or mean. Fisher’s exact test was used to compare differences in clinical variables between groups or classes. Associations between potential risk factors and clinical variables were assessed by Spearman’s rank correlation coefficient. Survival analysis was performed using the Kaplan-Meier method. The biological importance of the genetic mutated genes in different subtypes or classes was evaluated by KEGG pathway enrichment analysis using ClusterProfiler. A two-tailed P-value of < 0.05 was considered statistically significant. Statistical analysis was performed using SPSS 22.0 for Windows (SPSS, Chicago, IL, USA). Figures were created using GraphPad Prism (version 7.0; GraphPad, San Diego, CA) and the R statistical package v.3.5.1 (http://www.r-project.org/).

## Results

### Patient Cohort Characteristics

**Figure 1:**
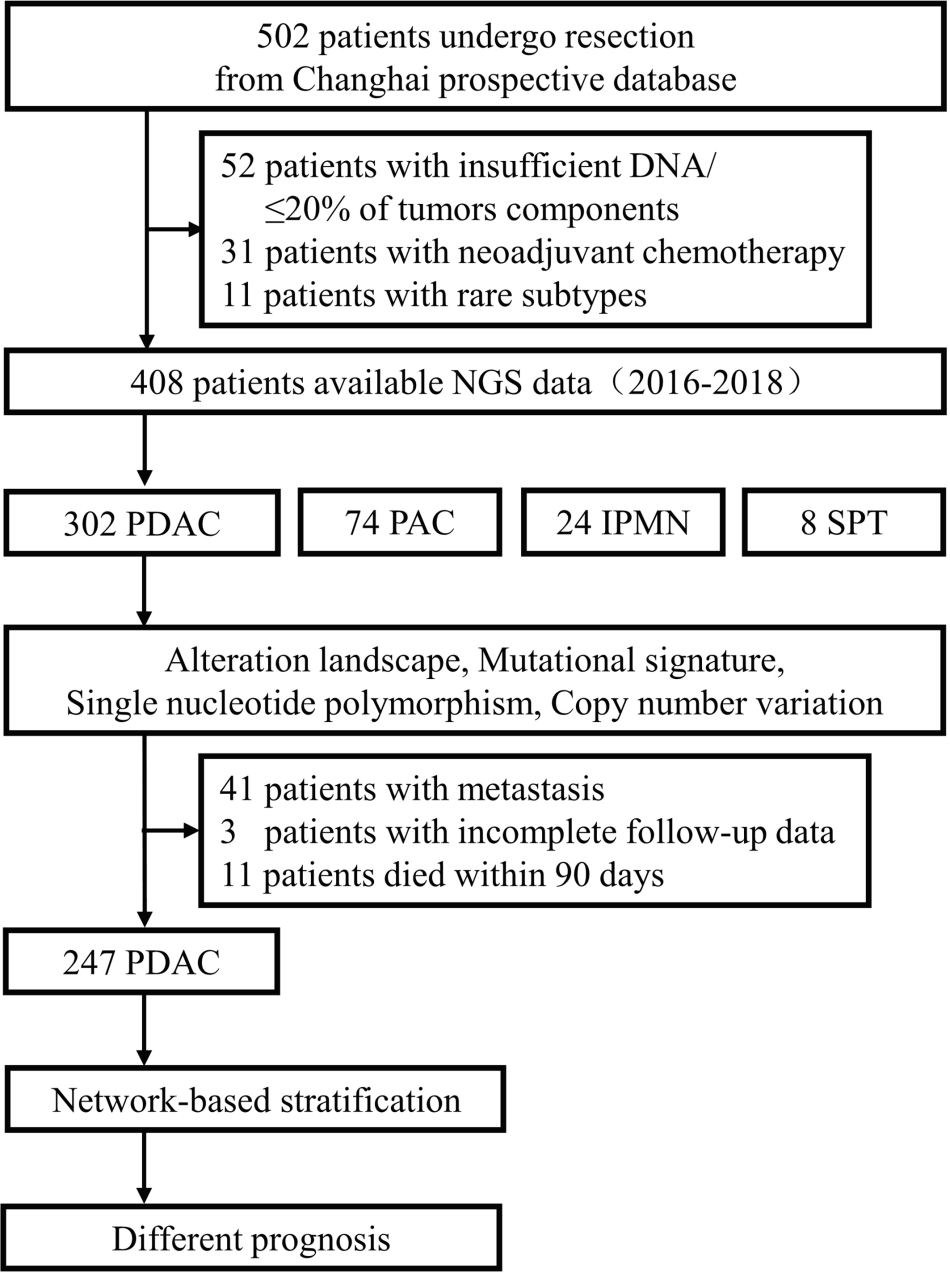
Workflow diagram of the study.

A total of 502 pancreatic and periampullary neoplasm specimens were submitted for targeted genomic profiling during clinical care. Four hundred and fifty samples passed pre- and post-sequencing quality control, and most of the failures were due to less than 20% of tumor components or insufficient DNA. After further removing 31 samples from patients who had neoadjuvant therapy as well as 11 samples diagnosed as rare subtypes, 302 cases of pancreatic duct adenocarcinoma (PDAC), 74 cases of periampullary carcinoma (PAC), 24 cases of intraductal papillary mucinous neoplasm (IPMN), and 8 cases of solid-pseudopapillary tumor (SPT) had credible sequencing results (**Figure 1**). Among the 302 PDAC patients, 199 were males and 103 were females that ranged in age between 33 and 86 years (mean, 60.9 years; median, 62.0 years) at the time of targeted genomic profiling. Two hundred and sixty-one samples were from radical resection specimens, and 41 samples were from preoperative or intraoperative FNA biopsy specimens. Sixty-eight patients were in stage I of AJCC 8th, 158 in stage II, 35 in stage III, and 41 in stage IV. The median overall survival time for the 41 patients with metastasis was 7.1 months (95% CI: 4.102–10.098). After excluding 11 cases that died within 90 days of surgery and 3 cases without complete follow-up data, the remaining 247 patients who underwent radical resection had a median overall survival time of 21.3 months (95% CI: 19.138–23.462). The median follow-up time for radical resection and metastatic patients was 15.0 months and 5.9 months, respectively.

### Genomic Landscape of PDAC, PAC, IPMN, and SPT Genetic Alterations

**Figure 2:**
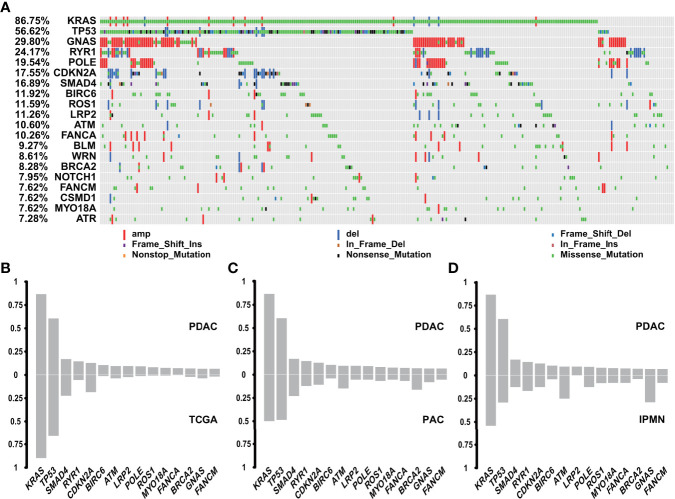
Genomic landscape of genetic alterations in PDAC and comparison with other subtypes. **(A)** Genomic landscape of PDAC genetic alterations (n = 302). **(B)** Comparison between PDAC (n = 302) and TCGA database (QCMG, n = 383) in genetic mutations. **(C)** Comparison between PDAC (n = 302) and PVC (n = 74) in genetic mutations. **(D)** Comparison between PDAC (n = 302) and IPMN (n = 24) in genetic mutations.

Among the 302 PDACs, a total of 19,120 genomic genetic alterations, including SNV, InDel, and CNV, were identified in 317 genes. The most frequent genomic alteration in PDAC was KRAS (n = 262, 86.75%), followed by TP53 (n = 171, 56.62%), GNAS (n = 90, 29.80%), RYR1 (n = 73, 24.17%), and POLE (n = 59, 19.54%) (**Figure 2A**). The alteration rates of two common driver genes, CDKN2A and SMAD4, were 17.55% (n = 53) and 16.89% (n = 51), respectively (**Figure 2A**). Comparison with public data (QCMG, 2016) showed similar results in the four driver genes but a higher alteration rate in genes like RYR1, BIRC6, ATM, LRP2, and POLE (**Figure 2B**).

Genomic alterations of PAC occurred in TP53 (n = 39, 52.70%), KRAS (n = 38, 51.35%), MUC17 (n = 26, 35.14%), GNAS (n = 23, 31.08%), and CABIN1 (n = 19, 25.68%), in decreasing order (**Supplementary Figure 1A**). Among the top 15 mutated genes in PDAC, PAC had lower rates in TP53 and KRAS, but was enriched in DNA damage repair (DDR)-related genes, including BRCA2 and ATM (32, 33) (**Figure 2C**), which indicated a different driving origin and more treatment options. The genomic alteration landscape of 24 cases of IPMN was characterized by GNAS (n = 13, 54.17%), KRAS (n = 13, 54.17%), MUC17 (n = 11, 45.83%), POLE (n = 9, 37.50%), and TP53 (n = 7, 29.17%) (**Supplementary Figure 1B**). Compared with PDAC, GNAS was another common driver gene for IPMN, aside from KRAS and TP53. In addition, ATM mutations in IPMN accounted for 20.83% in our cohort, which is much higher than in PDAC (**Figure 2D**). SPT lacked alterations in genes commonly found in PDAC, PAC, and IPMN. The molecular hallmark of SPT was represented by mutations in the CTNNB1 gene (**Supplementary Figure 1C**), which is involved in the Wnt/β-catenin signaling pathway and reportedly observed in more than 90% cases (34). Further function analysis revealed that PDAC, PAC, and IPMN had comparable changes in the KEGG signaling pathways, while SPT showed fewer pathways with abnormalities (**Supplementary Figure 1D**).

### Mutational Signature of PDAC

**Figure 3:**
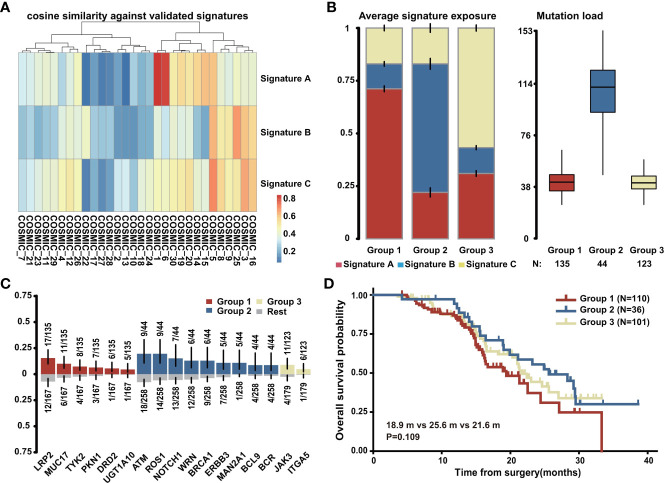
Mutational signature of PDAC. **(A)** The correlation between three identified mutational signatures in PDAC (n = 302) and reported COSMIC signatures. **(B)** Three groups of PDAC patients (n = 302) classified by average signature exposure (left) and their mutation load (right). **(C)** Specific gene mutations enriched in the three groups (n = 302). **(D)** Overall survival analysis of the three groups with PDAC patients who received radical resection and had complete follow-up data (n = 247).

Three mutational signatures (A, B, and C) of all 302 PDAC samples were identified *via* the NMF method and were highly similar to the reported COSMIC signatures: COSMIC_1, COSMIC_25, and COSMIC_5, respectively (**Figure S2A**; **Figure 3A**). The etiology of signatures 25 and 5 remain unknown, while signature 1 was thought to be the result of an endogenous mutational process initiated by spontaneous deamination of 5-methylcytosine (**Supplementary Figure 2A**). Based on signature exposure, we divided 302 samples into three groups, which were separately enriched for signatures A, B, and C (**Figure 3B**). Further analysis of mutation load revealed that there was a notably higher mutation load in group 2 compared with the other two groups (**Figure 3B**). Genes like ATM, ROS1, NOTCH1, BRCA1, WRN, ERBB3, MAN2A1, BCL9, and BCR were more commonly mutated in group 2 (**Figure 3C**). Statistical comparison of clinicopathological characteristics between the three groups suggested that only diabetes was related to different mutational signatures (**Supplementary Table 2**); the number of patients with diabetes mellitus was relatively high in groups 1 and 3 (**Supplementary Figure 2B**). We matched the mutational spectrum of diabetes cases with cases that did not have diabetes and found that the diabetes cases exhibited strand bias for C > A mutations at T [C > A] A, while the no-diabetes cases exhibited strand bias for C > G mutations at T [C > G] C and T > G mutations at C [T > G] G (**Supplementary Figure 2C**), indicating potential heterogenous carcinogenic factors in PDAC. Further survival analysis of the 247 PDAC patients who received radical resection showed that group 2 with more mutations in DDR-related genes and less diabetes mellitus had a trend for better prognosis among the three groups (**Figure 3D**).

### Genetic Mutation in PDAC

**Figure 4:**
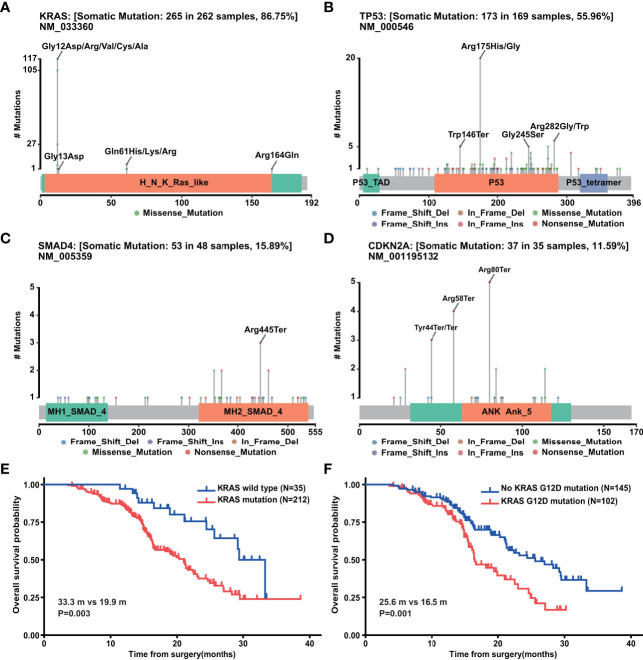
Genetic mutation of PDAC. **(A)** Mutation sites of KRAS gene in PDAC (n=302). **(B)** Mutation sites of TP53 gene in PDAC (n = 302). **(C)** Mutation sites of SMAD4 gene in PDAC (n = 302). **(D)** Mutation sites of CDKN2A gene in PDAC (n = 302). **(E)** Overall survival analysis of above 247 PDAC patients with (n = 212) and without (n = 35) KRAS mutations. **(F)** Overall survival analysis of above 247 PDAC patients with (n = 102) and without (n = 145) KRAS G12D mutation.

The most commonly altered gene, KRAS, had 265 mutations in 262 samples, in which 117 cases were G12D, 105 cases were G12V, 27 cases were G12R, 5 cases were G12C, and 1 case was G12A (**Figure 4A**). Besides codon 12, mutations also occurred in codons 13, 61, and 164 including G13D, Q61H, Q61K, Q61R, and R164Q (**Figure 4A**). TP53 was detected 173 mutations in 169 samples (**Figure 4B**). The most frequently mutated site lay in codons 175 (n = 20), 282 (n = 6), and 245 (n = 5) (**Figure 4B**). Another two tumor suppressors, SMAD4 and CDKN2A, had 53 mutations in 48 samples and 37 mutations in 35 samples, respectively (**Figures 4C, D**). We next explored the correlation between the top 20 mutated genes and clinicopathological characteristics (**Supplementary Figure 3A**). KRAS mutation was associated with T stage as well as hypertension. TP53 mutation was related to differentiation degree. CDKN2A mutation correlated with drinking history and perineural invasion, while no clinical variable was associated with SMAD4 mutation. GNAS, WRN, and LRP2 mutations were related to T stage, N stage, and M stage, respectively. No gene was found to be associated with more than two clinical variables, while perineural invasion showed a strong association with the most genes including WRN, CDKN2A, BRAC2, and BLM. Further survival analysis of four PDAC driver genes with the 247 patients above displayed that mutation of KRAS, especially KRAS G12D (**Figures 4E, F**), but not TP53, CDKN2A, or SMAD4 was indicative of prognosis (**Supplementary Figures 3B–D**).

### Copy Number Variation in PDAC

**Figure 5:**
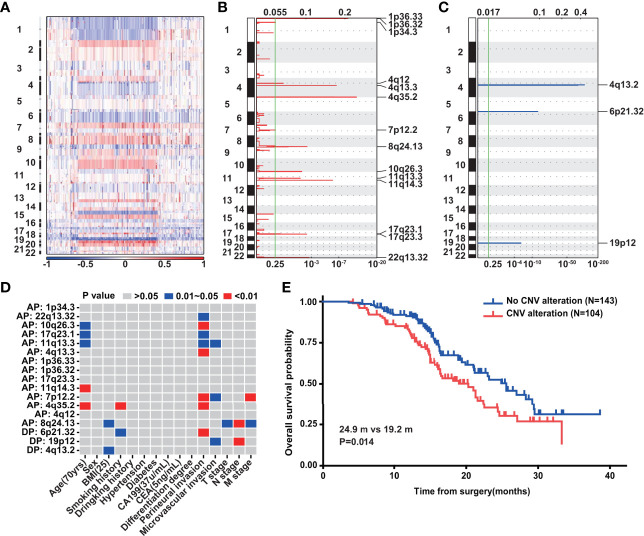
Copy number variation of PDAC. **(A)** The landscape of CNV in PDAC (n = 302). **(B)** Gains of CNV in PDAC (n = 302). G-score at top and q-value at bottom. **(C)** Losses of CNV in PDAC (n = 302). G-score at top and q-value at bottom. **(D)** The correlation between CNVs and clinical variables in PDAC (n = 302). **(E)** Overall survival analysis of above 247 PDAC patients with (n = 104) and without (n = 143) the 17 significant chromatin structure alterations.

The CNV landscape of 302 PDAC cases is summarized in **Figure 5A**. The most striking amplification lay in chromosomes 1, 4, 7, 8, 10, 11, 17, and 22 (**Figure 5B**), while deletion existed mainly in chromosomes 4, 6, and 19 (**Figure 5C**). We extracted 17 significant structure alterations from the above chromosomes to examine their association with clinical variables (**Figure 5D**). Amplification of 8q24.13, which has been previously studied in gastric cancer (35) and breast cancer (36), was closely correlated with T, N, and M stage, simultaneously. Perineural invasion was also associated with the most chromosome fragment alterations including gains of 4q13.3, 4q35.2, 7p12.2, 10q26.3, 11q13.3, 17q23.1, and 22q13.32 as well as loss of 6p21.32. Local chromosomal transcriptional up-regulation at chromosome 4q13.3 was observed in the neuroectodermal conversion process of human mesenchymal stem cells (37). Additionally, the FAT1 (38) and TMEM16H (39) genes, which have been proved to be highly expressed in neural tissues, were contained in 4q35.2 and 11q13.3, respectively. Importantly, according to survival analysis with the 247 PDAC patients, we found that patients without any of the above 17 chromatin structure alterations had a markedly better prognosis (P = 0.014) (**Figure 5E**).

### Network-Based Stratification of PDAC With Genetic Mutations

**Figure 6:**
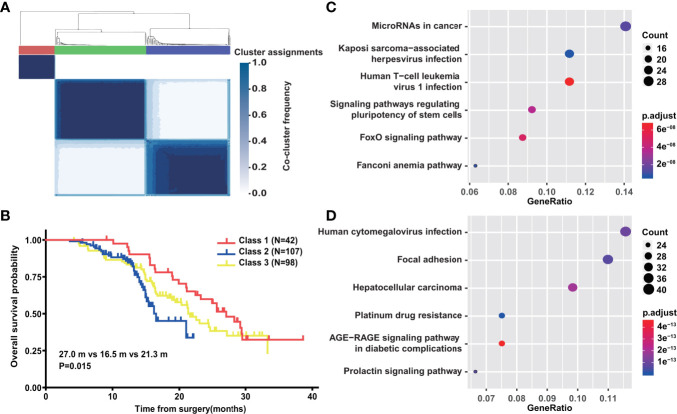
Network-based stratification of PDAC with genetic mutation. **(A)** Schematic of network-based stratification in above 247 PDAC (n = 247). **(B)** Overall survival analysis of above 247 PDAC patients classified by network-based stratification. **(C)** The differential KEGG signal pathways among top 30 significantly enriched in class 1. **(D)** The differential KEGG signal pathways among top 30 significantly enriched in class 2.

To systematically investigate the classification of PDAC with only genetic mutations, we used network-based stratification to produce a robust subdivision of three classes (**Figure 6A**). We next explored whether such stratification was of prognostic value. In the survival analysis of the 247 PDAC patients, a remarkable difference was observed among the three classes (P = 0.015) (**Figure 6B**). The median survival time of class 1 was 27.0 months (95% CI: 22.630–31.370), while the corresponding time of class 2 was only 16.5 months (95% CI: 12.102–20.898) (**Figure 6B**). KEGG gene set enrichment analysis was further performed to better unveil the underlying biology of class 1 and class 2. The differential pathways among the top 30 showed that genetic mutations involved in class 1 mainly focused on MicroRNAs in cancer, Kaposi sarcoma-associated herpesvirus infection, Human T-cell leukemia virus 1 infection, signaling pathways regulating pluripotency of stem cells, the FoxO signaling pathway, and the Fanconi anemia pathway (**Figure 6C**). The Fanconi anemia pathway is a dedicated pathway for the repair of DNA interstrand crosslinks (40), which may partially explain the better prognosis of class 1. Additionally, human cytomegalovirus infection, Focal adhesion, Hepatocellular carcinoma, Platinum drug resistance, the AGE−RAGE signaling pathway in diabetic complications, and the Prolactin signaling pathway were enriched in class 2 (**Figure 6D**). Platinum drug resistance indicated a normal function of DDR in contrast to class 1. Furthermore, we compared clinicopathological characteristics between class 1 and class 2 (**Table 1**). Statistical analysis revealed that there was a higher proportion of diabetes mellitus in class 2, corresponding to the enriched pathway-AGE−RAGE signaling pathway in diabetic complications. Moreover, we found that class 1 was more inclined to be medium/well differentiated than class 2, which might result from differential molecular features.

**Table 1:** Comparison of clinicopathological characteristics between class1 and class2 in spectral clustering.

Variable	Class1(N = 42)	Class2(N = 107)	P value
Sex (N=149)			0.261
Female (%)	19 (45.2%)	37 (34.6%)	
Male (%)	23 (54.8%)	70 (65.4%)	
Age at surgery, y (N=149)			0.617
<70 (%)	37 (88.1%)	90 (84.1%)	
≥70 (%)	5 (11.9%)	17 (15.9%)	
BMI (N=143)			0.401
≤25 (%)	28 (70.0%)	79 (76.7%)	
>25 (%)	12 (30.0%)	24 (23.3%)	
Smoking history (N=146)			0.450
No (%)	29 (69.0%)	64 (61.5%)	
Yes (%)	13 (31.0%)	40 (38.5%)	
Drinking history (N=146)			0.817
No (%)	35 (83.3%)	83 (79.8%)	
Yes (%)	7 (16.7%)	21 (20.2%)	
Diabetes mellitus (N=146)			0.028
>No (%)	35 (83.3%)	67 (64.4%)	
Yes (%)	7 (16.7%)	37 (35.6%)	
Hypertension (N=146)			0.085
No (%)	32 (76.2%)	62 (59.6%)	
Yes (%)	10 (23.8%)	42 (40.4%)	
CEA at diagnosis, ng/mL (N=143)			0.237
Normal (%)	30 (75.0%)	65 (63.1%)	
Elevated (%)	10 (25.0%)	38 (36.9%)	
CA19-9 at diagnosis, U/mL (N=149)			0.827
Normal (%)	10 (23.8%)	23 (21.5%)	
Elevated (%)	32 (76.2%)	84 (78.5%)	
Differentiation degree (N=149)			0.021
Poor (%)	9 (21.4%)	42 (39.3%)	
Medium/Well (%)	33 (78.6%)	65 (60.7%)	
Perineural invasion (N=149)			1.000
>No (%)	3 (7.1%)	9 (8.4%)	
Yes (%)	39 (92.9%)	98 (91.6%)	
Microvascular invasion (N=149)			0.131
No (%)	31 (73.8%)	63 (58.9%)	
Yes (%)	11 (26.2%)	44 (41.1%)	
T stage (N=149)			0.053
T1	4 (9.5%)	8 (7.5%)	
T2	9 (21.4%)	45 (42.1%)	
T3/T4	29 (69.0%)	54 (50.5%)	
N stage (N=149)			1.000
N0	17 (40.5%)	43 (40.2%)	
N1/N2	25 (59.5%)	64 (59.8%)	

PDAC, pancreatic ductal adenocarcinoma; BMI, body mass index; CEA, carcinoembryonic antigen; CA19-9, carbohydrate antigen 19-9.

## Discussion

Pancreatic cancer is one of the most lethal diseases worldwide, but etiology, diagnosis, therapeutic modality, and prognosis vary in different regions and races, especially between Asian, European, and American countries. It has been reported that blacks have a higher incidence of pancreatic cancer than other racial/ethnic groups (41, 42). As an unequivocal risk factor for pancreatic cancer (43), smoking habits differ significantly among racial/ethnic groups. Asian females have the lowest smoking rates, whereas Asian men have the second lowest rate after Hispanic men, compared with other racial/ethnic groups (44). Furthermore, diabetes mellitus, which is an associated factor of pancreatic cancer, has a markedly different incidence around the world (3, 45, 46). These factors could lead to distinct origins of pancreatic carcinogenesis, which might cause variations in genomic alterations, clinicopathological features, and prognosis. In fact, oncologists in different countries prefer to use different drug compounds. For example, researchers in the United States are more inclined to use FORFIRINOX (47), while European researchers used Gemcitabine plus Capecitabine (48), and Asian researchers are more willing to use S1 (49). This may be the result of diverse pharmacogenomic profiles. However, at present, there is no clear consensus on this issue, especially in Oriental patients. This study is the first to report genetic alteration profiles from the largest target sequencing cohort of Oriental pancreatic cancer patients. A large number of specific mutations related to several specific clinicopathological factors were identified in Oriental PDAC patients. Importantly, the relationship between genetic alterations and prognosis in PDAC was based on high-quality data from a standardized treatment center for pancreatic cancer, and the data were well analyzed to demonstrate critical genomic impacts on tumor biological behavior. Our findings will help to optimize clinical treatment decisions of Oriental PDAC patients and improve their survival time.

KRAS, TP53, CDKN2A, and SMAD4 are the four major driver genes mutated in nearly 100% of PDAC patients (50). Their functions have been widely investigated. KRAS is considered a driver gene in the initial stages of cancer, and its reported mutation rate varied from 72% to 90% (18, 51, 52). However, further research showed that the low mutation frequency was likely caused by low tumor cellularity or insufficient sequencing depth, which could be compensated using laser microdissection or increasing the depth of sequencing. In this study, well-designed targeted capture probes and high depth sequencing (>1000X) were used to reveal the true frequency of the 436 cancer-related genes. In our cohort, the KRAS mutation rate was 86.75%, which is similar to the results in a public database of patients undergoing microdissection (18) or with high tumor cellularity (52). Alternatively, common sites including 12, 13, and 61 in 12 KRAS mutation negative samples (all from FNA biopsy specimens) were validated by ddPCR (data not shown). Three samples were found to contain a low frequency of KRAS mutation, which implied low tumor purity. Although studies have reported that enough samples can be obtained *via* FNA for sequencing analysis (53), it is difficult for pancreatic cancer due to the influence of tumor location, puncture bleeding, puncture proficiency, and tumor purity. However, this result is very important for pancreatic cancer patients since most have advanced disease, and a positive result for KRAS mutation will determine the clinical treatment strategy. The serious impact of KRAS mutation on survival reminds that clinicians should interpret and handle the negative results with extreme caution.

TP53 is one of the most frequently mutated tumor suppressor genes in diverse cancers. However, the mutation frequency in public database of pancreatic cancer scattered, which included data from different countries and ethnic groups. The lowest frequency data were reported in 2012, with a mutation frequency of only 33% (54). The highest group was reported in 2017 and came from 149 samples with a mutation frequency of 72% (17). Most of the other cohorts ranged between 50% and 60% (18, 51). Our study identified a mutation frequency of 55.96% in common mutation forms. The driver genes SMAD4 and CDKN2A have two main inactivation subtypes: mutation and copy number depletion. The mutation rates in our cohort were 15.89% and 11.59%, respectively, similar to previous studies (51). However, the copy number deletion fluctuated widely from previous results due to different sequencing and bioinformatic analysis methods. Paired sequencing, whole-exome sequencing (WES), and whole-genome sequencing (WGS) generally had higher sensitivity for detecting CNVs. The detection rate of CDKN2A deletion reached nearly 40% using WGS (55), while only 10% or lower was able to be detected in panel sequencing of single sample.

Interestingly, in our Oriental cohort, the mutation frequency of genes related to DDR was significantly higher than previously reported. For instance, ATM (DNA-damage response gene) mutation frequency in our cohort was 9.60%, much higher than ICGC (1.0%), TCGA PanCancer Atlas (4.5%), and in other studies (3%–5%) (18, 52), indicating a significant difference between Eastern and Western populations. Other DDR genes including POLE (homologous DNA pairing and strand exchange), FANCA (interstrand DNA cross-link repair), BLM (DNA double-strand break repair), WRN (DNA double-strand break repair), BRCA2 (homologous DNA pairing), FANCM (DNA double-strand break repair), and ATR (DNA damage sensor) had higher mutation rates in our cohort. Such results mean that Oriental pancreatic cancer patients may have different etiologies and cancer development processes. Furthermore, other important genes related to cancer treatment including CSMD1 (potential suppressor of squamous cell carcinomas), BRIC6 (apoptosis-associated gene), and ROS1 (the member of tyrosine kinase insulin receptor genes) were more enriched in this study. These findings might partially explain the different effects of PARP inhibitors, platinum, and fluorouracil drugs in Asians and promote their further application.

To investigate possible etiologies, we further analyzed mutational signature and found some interesting results. Using NMF, we identified three novel mutational signatures (A, B, and C) that were highly similar to the reported COSMIC signatures 1, 25, and 5, respectively. Signature B dominant patients (group 2) had a markedly higher mutation load and were more enriched in mutations of genes associated with DDR, such as ATM, BRCA1, and WRN. These patients exhibited a trend of better prognosis than the other groups; however, this was not significant, which might be due to the infrequent use of platinum drugs as adjuvant therapy in this study. Clinically, group 2 had less diabetes mellitus, which indicated that there were more C > G mutations at T [C > G] C and more T > G mutations at C [T > G] G but fewer C > A mutations at T [C > A] A. An emerging idea is that altered metabolic status could contribute to cancer-causing mutations. It has been confirmed that long-term diabetes mellitus is a well-established risk factor of pancreatic cancer (56). Recent research illustrated that high glucose can trigger nucleotide imbalance and induce KRAS mutation in pancreatic cells (9). Consequently, we can speculate that long-term diabetes and prediabetes, rather than new-onset diabetes, is more likely to promote different genetic changes and lead to heterogeneity in carcinogenesis.

High-quality clinical data from a standardized treatment center for pancreatic cancer showed that the relationship between genetic alterations and clinicopathological factors is credible and worthy of discussion. In this study, we included the top 20 mutated genes (≥5.96%, 18 samples) for comparison due to their high reliability and strong clinical impact. Smoking is a well-recognized risk factor for pancreatic cancer. Approximately 20–25% of pancreatic cancers are attributed to cigarette smoking (57, 58). However, in this study, we found that smoking was unrelated to any type of genetic mutation, including genes previously thought to be related to tobacco. Previous studies showed that the risk of developing pancreatic cancer decreased rapidly after a few years of smoking cessation, and may even be reduced to the level of never smokers after 15–20 years of smoking cessation (57–59). This reversible risk suggests that smoking does not promote the development of pancreatic cancer by inducing genetic mutations. Therefore, the carcinogenic effect is more likely to involve transcriptomics or epigenetics, which deserves more attention. Perineural invasion, a common pathological feature of cutaneous squamous cell cancer (CSCC), head and neck squamous cell carcinoma (HNSCC), and PDAC, predicted poor survival (60–62). A previous study reported that cancer cell proliferation is a common response to neural cancerous microenvironments (63). Furthermore, single-cell transcription data analysis showed that perineural invasion relevant module genes were associated with EMT, invasion, and metastasis (64). Moreover, the overall proteomic profiles between perineural invasion and non-perineural invasion tumor samples appeared largely similar (65). Therefore, perineural infiltration is more likely a marker for the changes in tumor microenvironment than directly correlated with genomic alterations, and we can reasonably infer that pancreatic cancer cells with perineural invasion-associated mutations and CNVs in this study might be more invasive.

It is very important to establish the relationship among gene mutations, treatment, and prognosis, especially for driver or key genes, since this may suggest targets for more effective drugs in clinical practice such as EGFR in lung cancer (66) and KIT in gastrointestinal stromal tumors (67). However, accurate correlation analysis when dealing with multiple mutation forms and sites is difficult. Furthermore, due to complex gene functions within a network, interactions can affect the analysis of a specific gene, while block analysis without dimension reduction can result in overfitting. Therefore, appropriate methodology is critical for the analysis of mutation and prognosis. A variety of methods were used in this study, among which network-based stratification (31) showed the best results. Using network knowledge, we stratified the cohort of PDAC patients into three robust classes with genetic mutations only, which were biologically informative and had a strong association with clinical outcomes such as patient prognosis and drug sensitivity. Survival analysis revealed that class 1 had significantly better overall survival than class 2 and class 3. Furthermore, there was enrichment of the DDR-relevant Fanconi anemia pathway in class 1, indicating a recommendation for platinum drugs (40), while platinum drug resistance was observed in class 2 due to a higher rate of poor differentiation and diabetes mellitus. Application of such classification strongly facilitated the understanding of underlying genomic heterogeneity of PDAC, which may help to stratify patients and optimize individualized treatments in clinical practice.

However, our study had limitations. The study was retrospective and therefore may have included some bias. The conclusions made from this research should be verified in a prospective study. In addition, sequencing only tumor samples restrained the analysis of germline mutations in pancreatic cancer, which requires further characterization.

In summary, we report here the largest sequencing cohort and describe the genetic alteration landscape in Oriental pancreatic cancer patients for the first time. Large numbers of specific mutations were identified in Oriental PDAC samples and were closely related to several clinicopathological factors. Furthermore, under the premise of a standardized treatment center, the relationship between genetic alterations and prognosis in PDAC was analyzed to delineate the important genomic impact on tumor biological behavior. This research will help to optimize clinical treatment decisions of Oriental PDAC patients and improve their survival time.

## Conclusions

In this study, we reported for the first time the genetic alteration landscape of Oriental PDAC patients identifying many Oriental-specific alterations. The relationship between genetic alterations and clinicopathological factors as well as prognosis demonstrated important genomic impact on tumor biology. This study will help to optimize clinical treatment of Oriental PDAC patients and improve their survival.

## Data Availability Statement

The data presented in the study are deposited in the National Infrastructure of Chinese Genetic Resources repository (https://202.108.211.75/#/main/project?xmid=4280), accession number (BF2021030304684).

## Ethics Statement

The studies involving human participants were reviewed and approved by the Shanghai Changhai Hospital Ethics Committee. The patients/participants provided their written informed consent to participate in this study.

## Author Contributions

SWG, XS, SZG, GL, and GJ contributed to conception of the study. SWG, XS, SZG, QH, LJ, and LL designed the experiments. SWG, XS, SZG, QH, and LJ performed the experiments. BL, JS, HW, SS, GZ, YP, BL, WL, XX, KZ, ZS, and WJ provided all the clinical information. SWG, XS, SZG, GL, GJ analyzed all the data. SWG, XS, and SZG wrote the manuscript. GL and GJ revised the manuscript. All authors contributed to the article and approved the submitted version.

## Funding

This study was partially supported by National Natural Science Foundation of China (81972913 and 82002559), National Key Research and Development Project (2019YFC1315904), Shanghai Municipal Natural Science Foundation (20ZR1456500), The “234 Discipline Climbing Plan” Project of the First Affiliated Hospital of Naval Military Medical University (2019YXK033) and The Youth Startup Fund of the First Affiliated Hospital of Naval Medical University (2019QNA07).

## Conflict of Interest

Authors QH, LJ and LL were employed by the company Shanghai Biotecan Medical Diagnostics Co., Ltd.

The remaining authors declare that the research was conducted in the absence of any commercial or financial relationships that could be construed as a potential conflict of interest.

## Supplementary Material

Supplementary Figure 1
**Genomic landscape of somatic alterations in other subtypes.** (A) Genomic landscape of PVC somatic alterations (n=74). (B) Genomic landscape of IPMN somatic alterations (n=24). (C) Genomic landscape of SPT somatic alterations (n=8). (D) KEGG signal pathway analysis of IPMN, PVC, PDAC and SPT.

Supplementary Figure 2
**Mutational signature of PDAC.** (A) Three mutational signatures identified by NMF analysis and their highly similar reported COSMIC signatures. (B) Comparison of diabetes mellitus status in the three groups of PDAC patients (n=302). (C) Comparison of mutational spectrum between PDAC patients with (n=80) and without (n=216) diabetes mellitus.

Supplementary Figure 3
**Somatic mutation of PDAC.** (A) The correlation between somatic mutations and clinical variables in PDAC (n=302). (B) Overall survival analysis of above 247 PDAC patients with (n=144) and without (n=103) TP53 mutations. (C) Overall survival analysis of above 247 PDAC patients with (n=39) and without (n=208) SMAD4 mutations. (D) Overall survival analysis of above 247 PDAC patients with (n=29) and without (n=218) CDKN2A mutations.
