# Supplementary material for: The Landscape of Genetic Alterations Stratified Prognosis in Oriental Pancreatic Cancer Patients
Source: Front Oncol. 2021 Jul 22;11:717989. doi: 10.3389/fonc.2021.717989 (PMC8340855; doi:10.3389/fonc.2021.717989)
Supplement: Supplementary file 1 [file Table_1.doc]

**Table S1. Panel list of 436 cancer-related genes.**

| ABCG2 | AXL | CHRM1 | ESRRG | GRM5 | KRAS |
| --- | --- | --- | --- | --- | --- |
| ABL1 | BAP1 | CSF1R | ETV1 | GSR | LCK |
| ABL2 | BCL2 | CSNK2A1 | ETV4 | GSTP1 | LHCGR |
| ACVR2A | BCR | CTNNB1 | ETV5 | HGF | LMNA |
| ACVRL1 | BIRC2 | CXCR4 | EWSR1 | HMGCR | LTA4H |
| ADA | BIRC3 | CYP11A1 | EXT1 | HNF1A | LYN |
| ADAM17 | BIRC6 | CYP11B1 | EXT2 | HOXB13 | MAN2A1 |
| ADAM28 | BIRC7 | CYP17A1 | EZH2 | HRAS | MAP2K1 |
| ADAMTS5 | BLM | CYP19A1 | EZR | HSP90AA1 | MAP2K2 |
| ADORA3 | BMPR1A | CYP1B1 | F2 | HSP90AB1 | MAP2K3 |
| ADRA1A | BRAF | DDB1 | FANCA | HSP90B1 | MAP2K4 |
| ADRA1B | BRCA1 | DDR1 | FANCM | HTR1A | MAP2K5 |
| ADRA2A | BRCA2 | DDR2 | FBXW7 | HTR1B | MAP2K6 |
| ADRA2B | BRIP1 | DHODH | FGFR1 | HTR1D | MAP2K7 |
| ADRA2C | BTK | DICER1 | FGFR2 | HTR2A | MAPK1 |
| AKAP9 | C1R | DNMT3A | FGFR3 | HTR2B | MAPK11 |
| AKR1B1 | C1S | DPYD | FGFR4 | HTR2C | MAPK12 |
| AKR1D1 | CA6 | DRD1 | FH | HTR7 | MAPK13 |
| AKT1 | CABIN1 | DRD2 | FLCN | IDH1 | MAPK14 |
| AKT2 | CAD | DRD3 | FLT1 | IDH2 | MAPK3 |
| AKT3 | CASP3 | DRD4 | FLT3 | IFITM3 | MAPK8 |
| ALDH1A1 | CBL | DRD5 | FLT4 | IGF1R | MAPK9 |
| ALDH1A2 | CCNE1 | EDNRA | FNTA | IMPDH1 | MAX |
| ALK | CCR4 | EGFR | FNTB | IMPDH2 | MCL1 |
| ALOX5 | CD44 | EGLN1 | FOLH1 | ITGA5 | MDM2 |
| ANGPT1 | CDC73 | EML4 | FOXL2 | ITGAL | MDM4 |
| ANGPT2 | CDH1 | EPCAM | FRK | ITGAV | MED12 |
| ANPEP | CDK2 | EPHA2 | FSHR | ITGB1 | MEN1 |
| APC | CDK4 | EPHB4 | FYN | ITGB3 | MERTK |
| APEX1 | CDK5 | EPRS | GATA2 | ITGB5 | METAP2 |
| APOL3 | CDK6 | ERBB3 | GATA3 | JAK1 | MLH1 |
| AR | CDK8 | ERBB4 | GFPT1 | JAK2 | MLH3 |
| ARAF | CDKN1A | ERCC3 | GNA11 | JAK3 | MMP1 |
| ATM | CDKN1B | ERCC4 | GNAQ | KCNH2 | MMP10 |
| ATP11B | CDKN2A | ERCC5 | GNAS | KCNH6 | MMP11 |
| ATR | CDKN2C | ERG | GNRHR | KCNH7 | MMP12 |
| AURKA | CFLAR | ESR1 | GPRC5A | KDR | MMP13 |
| AURKB | CHEK1 | ESR2 | GRIN1 | KIT | MMP14 |
| AURKC | CHEK2 | ESRRA | GRIN3A | KLK2 | MMP15 |
| MMP16 | NFKB2 | PIK3R2 | PSEN1 | SDC4 | TP53 |
| MMP17 | NOS2 | PIK3R3 | PSEN2 | SDHA | TRIM33 |
| MMP19 | NOTCH1 | PIK3R4 | PSMA8 | SERPINE1 | TRPM8 |
| MMP2 | NPM1 | PIK3R5 | PSMB10 | SF3B1 | TSC1 |
| MMP20 | NR0B1 | PIM1 | PSMC2 | SLC25A5 | TSC2 |
| MMP21 | NR3C1 | PKN1 | PSMC5 | SLC25A6 | TUBA1B |
| MMP24 | NRAS | PKN2 | PSMD7 | SLC2A2 | TUBA1C |
| MMP25 | NRG1 | PKN3 | PSMF1 | SLC3A2 | TUBA3C |
| MMP26 | NTRK1 | PLAU | PTCH1 | SLC5A1 | TUBA4A |
| MMP27 | NTRK2 | PLCL1 | PTEN | SLC5A2 | TUBA8 |
| MMP3 | NTRK3 | PLK1 | PTGS2 | SMAD4 | TUBB1 |
| MMP7 | P2RY2 | PMS1 | PTK2 | SMARCB1 | TUBB3 |
| MMP8 | PALB2 | PMS2 | PTK2B | SMO | TUBD1 |
| MMP9 | PARP1 | PNP | PTPN11 | SOX2 | TUBE1 |
| MPL | PARP2 | POLA1 | PTPRS | SPOP | TUBG1 |
| MPRIP | PAX5 | POLE | PYGM | SRC | TXNRD1 |
| MSH2 | PDE5A | PPARA | RAC1 | SSTR1 | TXNRD2 |
| MSH6 | PDGFRA | PPARD | RAD50 | SSTR2 | TYK2 |
| MSR1 | PDGFRB | PPARG | RAD51 | SSTR5 | TYMS |
| MST1R | PDK1 | PPP2R1A | RAF1 | STAT3 | TYR |
| MTAP | PDK2 | PRKAA1 | RARA | STK11 | U2AF1 |
| MTOR | PDK3 | PRKAA2 | RARB | STRN | VCL |
| MUTYH | PDK4 | PRKCA | RARG | SYK | VDR |
| MYC | PGGT1B | PRKCB | RB1 | TACR1 | VHL |
| MYCN | PGR | PRKCD | RET | TEC | WEE1 |
| MYD88 | PIK3C2A | PRKCE | RORA | TEK | WRN |
| MYO18A | PIK3C2B | PRKCG | ROS1 | TERT | WT1 |
| NAIP | PIK3C2G | PRKCH | RPS6KA3 | TET2 | XIAP |
| NBN | PIK3C3 | PRKCI | RPS6KB1 | TFE3 | XPC |
| NCOA4 | PIK3CA | PRKCQ | RRM1 | TMPRSS2 | XPO1 |
| NF1 | PIK3CB | PRKCZ | RXRA | TOP1 | YES1 |
| NF2 | PIK3CD | PRKD1 | RXRB | TOP1MT | ZNF217 |
| NFE2L2 | PIK3CG | PRKD2 | RXRG | TOP2A |  |
| NFKB1 | PIK3R1 | PRKD3 | RYR1 | TOP2B |  |

**Table S2. Comparison of clinicopathological characteristics among different mutational signature groups of PDAC.**

| **Variable** | **Group 1**  **(N=135)** | **Group 2**  **(N=44)** | **Group 3 (N=123)** | **P value** |
| --- | --- | --- | --- | --- |
| Sex (N=302)  Female (%)  Male (%) | 44 (32.6%)  91 (67.4%) | 16 (36.4%)  28 (63.6%) | 43 (35.0%)  80 (65.0%) | 0.842 |
| Age at surgery, y (N=302)  <70 (%)  ≥70 (%) | 117 (86.7%)  18 (13.3%) | 41 (93.2%)  3 (6.8%) | 101 (82.1%)  22 (17.9%) | 0.194 |
| BMI (N=288)  ≤25 (%)  >25 (%) | 104 (80.6%)  25 (19.4%) | 35 (81.4%)  8 (18.6%) | 86 (74.1%)  30 (25.9%) | 0.440 |
| Smoking history (N=296)  No (%)  Yes (%) | 82 (62.1%)  50 (37.9%) | 26 (59.1%)  18 (40.9%) | 73 (60.8%)  47 (39.2%) | 0.934 |
| Drinking history (N=296)  No (%)  Yes (%) | 104 (78.8%)  28 (21.2%) | 35 (79.5%)  9 (20.5%) | 94 (78.3%)  26 (21.7%) | 1.000 |
| Diabetes mellitus (N=296)  No (%)  Yes (%) | 91 (68.9%)  41 (31.1%) | 39 (88.6%)  5 (11.4%) | 86 (71.7%)  34 (28.3%) | 0.028 |
| Hypertension (N=296)  No (%)  Yes (%) | 86 (65.2%)  46 (34.8%) | 31 (70.5%)  13 (29.5%) | 78 (65.0%)  42 (35.0%) | 0.828 |
| CEA at diagnosis, ng/mL (N=288)  ≤5 (%)  >5 (%) | 79 (61.2%)  50 (38.8%) | 27 (62.8%)  16 (37.2%) | 78 (67.2%)  38 (32.8%) | 0.611 |
| CA19-9 at diagnosis, U/mL (N=299)  ≤37 (%)  >37 (%) | 27 (20.1%)  107 (79.9%) | 12 (27.3%)  32 (72.7%) | 26 (21.5%)  95 (78.5%) | 0.600 |
| Differentiation degree (N=284)  Poor (%)  Medium/Well (%) | 49 (38.9%)  77 (61.1%) | 10 (23.8%)  32 (76.2%) | 33 (28.4%)  83 (71.6%) | 0.102 |
| Perineural invasion (N=280)  No (%)  Yes (%) | 8 (6.4%)  117 (93.6%) | 1 (2.4%)  40 (97.6%) | 11 (9.6%)  103 (90.4%) | 0.320 |
| Microvascular invasion (N=280)  No (%)  Yes (%) | 84 (67.2%)  41 (32.8%) | 28 (68.3%)  13 (31.7%) | 90 (78.9%)  24 (21.1%) | 0.108 |
| T stage (N=280)  T1  T2  T3/T4 | 10 (8.0%)  52 (41.6%)  63 (50.4%) | 5 (12.2%)  12 (29.3%)  24 (58.5%) | 9 (7.9%)  44 (38.6%)  61 (53.5%) | 0.647 |
| N stage (N=279)  N0  N1/N2 | 65 (52.4%)  59 (47.6%) | 16 (39.0%)  25 (61.0%) | 56 (49.1%)  58 (50.9%) | 0.339 |
| M stage (N=299)  M0  M1 | 115 (85.8%)  19 (14.2%) | 38 (86.4%)  6 (13.6%) | 105 (86.8%)  16 (13.2%) | 0.973 |

PDAC, pancreatic ductal adenocarcinoma; BMI, body mass index; CEA, carcinoembryonic antigen; CA19-9, carbohydrate antigen 19-9
