# Supplementary figures and images for: The Landscape of Genetic Alterations Stratified Prognosis in Oriental Pancreatic Cancer Patients
Source: Front Oncol. 2021 Jul 22;11:717989. doi: 10.3389/fonc.2021.717989 (PMC8340855; doi:10.3389/fonc.2021.717989)

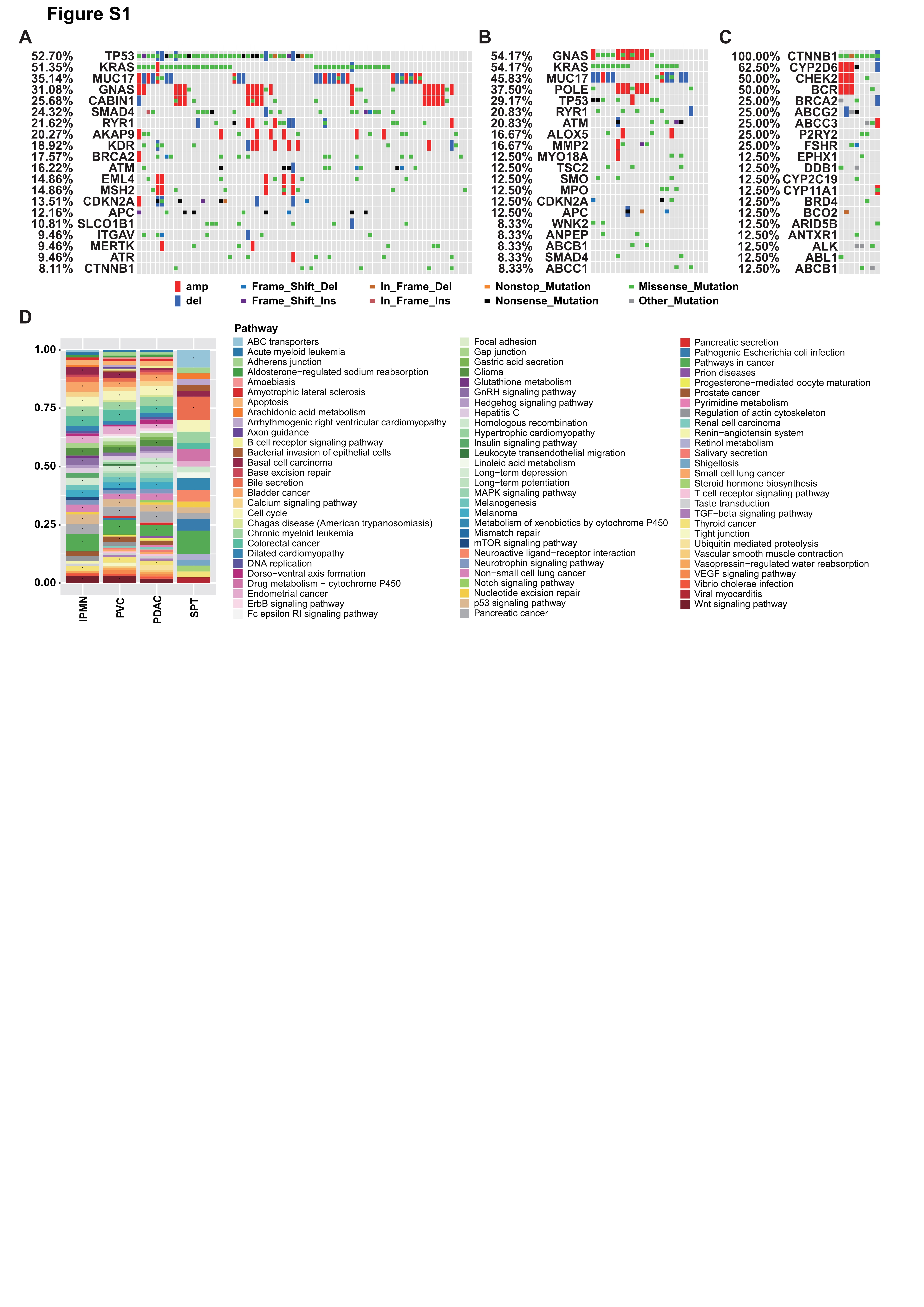

Supplement: Supplementary Figure 1 — Genomic landscape of somatic alterations in other subtypes. (A) Genomic landscape of PVC somatic alterations (n=74). (B) Genomic landscape of IPMN somatic alterations (n=24). (C) Genomic landscape of SPT somatic alterations (n=8). (D) KEGG signal pathway analysis of IPMN, PVC, PDAC and SPT. [file Image_1.jpeg]

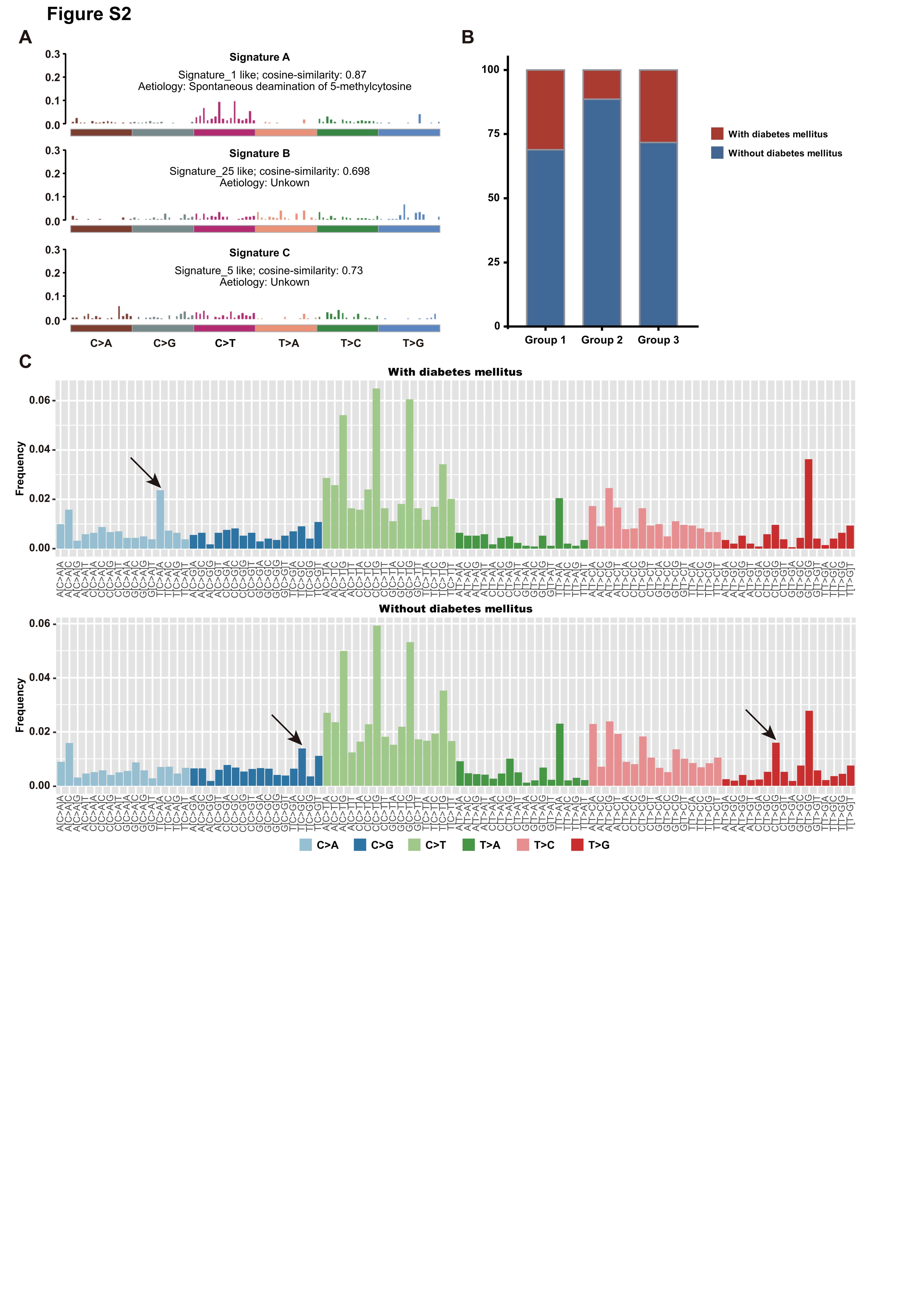

Supplement: Supplementary Figure 2 — Mutational signature of PDAC. (A) Three mutational signatures identified by NMF analysis and their highly similar reported COSMIC signatures. (B) Comparison of diabetes mellitus status in the three groups of PDAC patients (n=302). (C) Comparison of mutational spectrum between PDAC patients with (n=80) and without (n=216) diabetes mellitus. [file Image_2.jpeg]

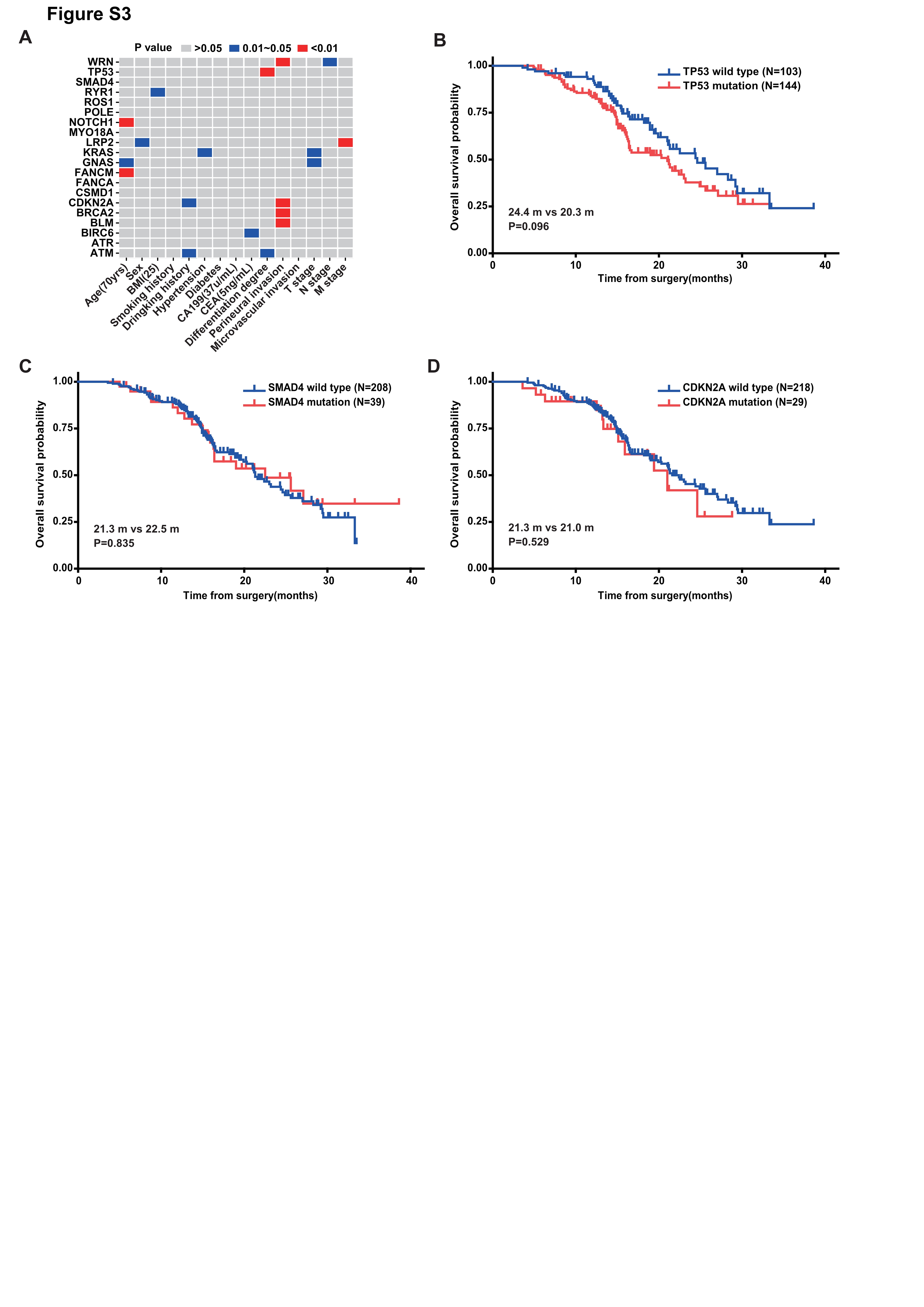

Supplement: Supplementary Figure 3 — Somatic mutation of PDAC. (A) The correlation between somatic mutations and clinical variables in PDAC (n=302). (B) Overall survival analysis of above 247 PDAC patients with (n=144) and without (n=103) TP53 mutations. (C) Overall survival analysis of above 247 PDAC patients with (n=39) and without (n=208) SMAD4 mutations. (D) Overall survival analysis of above 247 PDAC patients with (n=29) and without (n=218) CDKN2A mutations. [file Image_3.jpeg]
